# Age-related, interindividual, and right/left differences in anterior-posterior foot pressure ratio in preschool children

**DOI:** 10.1186/1880-6805-32-8

**Published:** 2013-04-20

**Authors:** Shigeki Matsuda, Shinichi Demura

**Affiliations:** 1 Gifu Shotoku Gakuen University, 1-38, Nakauzura, Gifu, Gifu, 500-8288, Japan; 2 Graduate School of Natural Science & Technology, Kanazawa University, Kakuma, Kanazawa, Ishikawa, 920-1192, Japan

**Keywords:** Anterior-posterior ratio, Children, Foot pressure, Infant

## Abstract

**Background:**

This study aimed to examine age-related, interindividual, and right/left differences in anterior-posterior foot pressure ratio in 764 preschool children (364 boys and 400 girls) aged 3.5-6.5 years.

**Methods:**

Subjects maintained an upright standing posture for 10 seconds on the Footview Clinic, an instrument designed to calculate the anterior-posterior foot pressure ratio. The ratio of anterior foot pressure in each subject’s right and left feet was selected as a variable, and the mean of a 10 s measurement was used for analysis.

**Results:**

The ratio of anterior foot pressure was significantly larger in the right foot than in the left foot. With regard to age, the ratio of anterior foot pressure was significantly larger in children aged over 4.5 years than in children aged 3.5 years. It was also larger in children aged 6 and 6.5 years than in children aged 4 years. Interindividual differences in variables were large, and coefficients of variance were highest in children aged 3.5 years and lowest in children aged 6.5 years.

**Conclusions:**

In conclusion, anterior foot pressure increases with age in preschool children. Interindividual differences in anterior foot pressure are large and tend to decrease with age. Furthermore, the anterior foot pressure is slightly higher in the right foot than in the left foot. These results will be useful for various studies, such as examining relationships between the anterior-posterior foot pressure ratio and factors, such as untouched toes, physical fitness, and level of exercise.

## Background

In recent times, the environment in which young children grow up has changed markedly in Japan. Various studies have examined the decrease in the physical fitness of Japanese children and contributing lifestyle factors, and countermeasures have been actively proposed. The phenomenon of untouched toes, where a child’s toes do not touch the floor while standing, has been reported as a concerning morphological change in young children [1-3], and a recent study reported an increased incidence of untouched toes in children [1,3]. The reasons for untouched toes and its effect on the body have been examined in many studies [4,5], and heel load has been noted as a reason for untouched toes [3]. It has been suggested that heel load induces a posterior inclination in the standing posture and causes the anterior part of the foot to float. Therefore, it is possible that foot pressure ratios increase with heel load in young children.

Some adverse effects of heel load have been reported. The position of the center of foot pressure moves forward from childhood to adulthood [6,7]. If a forward position is the norm, excessive heel load in childhood might contribute to problems in terms of growth and development. In addition, as the center of foot pressure changes, the standing posture also changes [8]. In short, it is believed that if heel load induces a posterior inclination in the standing posture, it will adversely affect standing posture. It has been reported that standing posture is most stable, from a mechanical perspective, when the anterior-posterior position of the center of foot pressure exists at approximately 40% of the foot length from the heel [8]. Therefore, there is a possibility that a posterior inclination in the standing posture caused by heel load increases the burden to muscle groups related to standing posture. It is important to remember that the center of gravity of the body moves forward during forward motions, such as walking and running. It is also possible that heel load triggers a delay of movement and increases consumption of energy at the start of movement, leading to inefficient walking and running.

As stated, it is suggested that heel load has a negative effect on postural stability. Therefore, the increase in the number of young children exhibiting heel load may be a serious problem. Recently, a device designed to measure the anterior-posterior foot pressure ratio was developed, facilitating easy determination of this ratio [9]. It is necessary to clarify the detailed actual condition of the anterior-posterior foot pressure ratio before examining the relationships between heel load, untouched toes, and so on. Although some studies have examined the anterior-posterior position of the center of foot pressure in adults [8,10], studies on young children are limited. Foot figure is formed because of the effects of various factors. Hence, data acquired from many young children with various circumstances will be needed. More accurate information on the formation of their foot figure will be obtained by the accumulation of various data. Although inter-trial reliability, sex differences in the anteriorposterior foot pressure ratio, and the influence of physical characteristics on this ratio have been examined [9], the effects of age on this ratio as well as interindividual and right/left differences have not been sufficiently examined. In particular, with regard to age difference in the anterior-posterior foot pressure ratio, it is desirable to examine the difference of a half-year interval, rather than a whole-year interval, in the case of young children with marked growth and development. By doing so, more appropriate knowledge on age difference will be obtained. With regard to interindividual differences, the differences in the anterior-posterior position of the center of foot pressure in adults have been previously examined [8]. However, there are few studies of this ratio in children. The detailed actual condition in the anteriorposterior foot pressure ratio in young children will be clear by clarifying interindividual difference (coefficients of variance, maximum, minimum, and range). In addition, the results of interindividual difference will be useful when determining discriminative values (outliers) in future studies and clinical setting.

Matsuda *et al*. [9] reported that the number of toes that touch the floor while standing increases every year in early childhood. Hence, it is hypothesized that the ratio of anterior foot pressure increases with age. However, there is a functional asymmetry in the human body [11]. Previc [12] reported that the left foot has a supporting function. Hence, right/left differences in anterior-posterior foot pressure ratios might also be present. This study aimed to examine age-related, interindividual, and right/left differences in anterior-posterior foot pressure ratio in preschool children. In addition, the anterior-posterior foot pressure ratios of adults were measured and the results for adults and children were compared, to clarify the characteristics in children.

The knowledge obtained in this study will provide basic data when examining the relationships between the anterior-posterior foot pressure ratio and problems occurring among children of this generation, such as untouched toes, decreased physical fitness levels, lack of physical activity, and deterioration of posture. In addition, this study will provide information for use in examining the detailed actual condition and variation in the anterior-posterior foot pressure ratio across all age groups, or for comparing the ratio between preschool children and other age groups in future studies.

## Methods

### Participants

Table 1 shows the number of participants and their physical characteristics. The participants were 867 preschool children aged 3.5-6.5 years (426 boys and 441 girls), who attended two kindergartens. Because 103 children (62 boys and 41 girls) could not conduct the measurement appropriately, they were excluded from the study prior to analysis. Hence, the participants who were analyzed were 764 children (364 boys and 400 girls). Children were recruited from two kindergartens located in Gifu and Yamagata cities, Gifu, Japan. The populations of Gifu (city) and Yamagata are approximately 410,000 and 30,000, respectively. It was considered that they did not have specific education policies, being different from other kindergartens in Japan. Participants were divided into six-month age ranges. For example, a participant who was 3.5 years or more but less than 4.0 years was classified into the 3.5 years old category, while a participant 4.0 years or more but less than 4.5 years was classified into the 4.0 years old category.

**Table 1:** The number of the participants and their physical characteristics

			**3.5 years old**	**4 years old**	**4.5 years old**	**5 years old**	**5.5 years old**	**6 years old**	**6.5 years old**	**Total**
Boys		*n*	31	72	57	52	51	74	27	364
	Height	Mean	98.7	101.7	104.4	107.8	111.1	115.0	116.5	107.9
	(cm)	SD	3.2	3.7	4.2	4.4	4.7	4.6	4.6	7.2
	Body mass	Mean	15.7	16.7	17.4	18.7	19.4	20.8	21.6	18.6
	(kg)	SD	1.6	1.7	1.8	2.3	2.4	2.9	2.4	2.9
Girls		*n*	32	76	72	52	76	64	28	400
	Height	Mean	97.9	100.7	103.9	107.5	110.7	113.4	115.4	106.9
	(cm)	SD	2.9	3.9	4.1	3.7	3.9	6.9	4.5	7.1
	Body mass	Mean	15.4	16.1	17.2	18.2	19.4	20.1	21.3	18.2
	(kg)	SD	1.4	1.7	2.1	2.1	2.4	2.1	2.8	2.7

SD, standard deviation.

The purpose of the study and the procedures to be undertaken were explained to the children’s parents in detail, and informed consent was obtained before the measurements. In addition, the children’s consent was obtained at the time of measurements. This experimental protocol was approved by the Ethics Committee on Human Experimentation of Faculty of Education, Kanazawa University.

In addition, the anterior-posterior foot pressure ratios of 37 young men (age: 18.6 ± 1.0 years, height: 171.1 ± 5.9 cm, body mass: 63.7 ± 9.1kg) were measured, to compare ratios between children and adults.

### Measurement device

**Figure 1:**
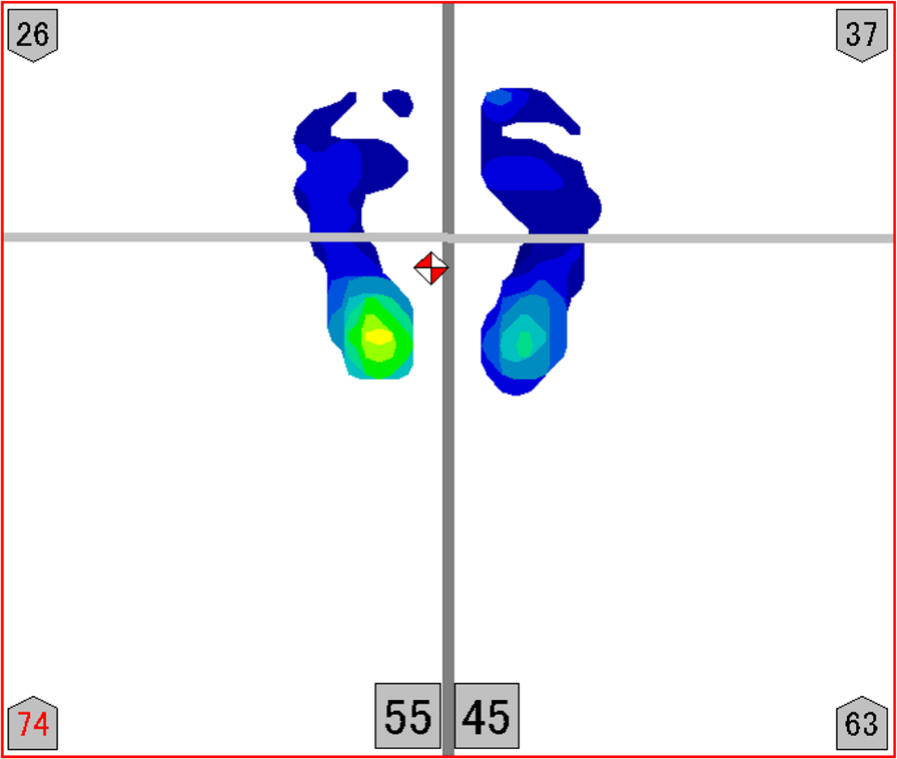
The anterior-posterior foot pressure ratio.

The Footview Clinic device (Nitta, Japan) was used to measure the anterior-posterior foot pressure ratio. This device is designed to calculate the anterior-posterior foot pressure ratio using all foot pressure obtained from the area of the foot that is in contact with the device in a standing position (Figure 1). The sampling frequency was 20 Hz. The anterior-posterior ratio refers to the pressure distribution between the anterior and posterior parts of the foot (in the left foot in Figure 1, pressure in the anterior part is 26% and pressure in the posterior part is 74%). Similar to a previous study [9], the line dividing the foot into anterior and posterior parts was set at the center of the foot length, which was defined as the distance from the back of the heel to the front of the longest toe. Considering the future development of the study in terms of reproduction of measurement, this basis, being straightforward and uncomplicated, was adopted. The foot length was calculated using a personal computer.

### Measurement procedure

The anterior-posterior foot pressure ratio was measured using a previously described method [9]. The measurement device was set horizontal to the floor. At that time, it was carefully confirmed that there was no slope of the floor, and no noise on the personal computer connected to the measurement device. The participants stood barefoot on the measurement device, with their feet 5 cm apart and their hands relaxed at their sides. They were instructed to look at a mark located at eye level and stabilize their posture for as long as possible during measurement. Before the measurement of the anterior-posterior foot pressure ratio, a stationary picture was recorded to obtain the foot length, which was necessary for analysis. A Footview Clinic device performed this step. Because some children had untouched-toes, a tester pushed the toe of the participant temporarily during the recording of the stationary picture. Subsequently, after confirming the postural stability of the participant, a 10 s measurement was initiated. Each participant was measured three times. It is important to note that some young children were unable to maintain a stable posture or could not continuously focus on the mark as instructed. These children (103 children) were excluded from the study before analysis.

### Evaluation variables

The ratio of anterior foot pressure in the right and left foot each was selected as a variable, and the mean of a 10 s measurement was used for analysis. As with a previous report, the second and third trials have high inter-trial reliability; therefore, these were used for analysis [9]. In addition, it was reported that there were no relationships between the ratio of anterior foot pressure in the right and left feet and physique (height and body mass) [9]. In this study, the correlation coefficients between the ratio of anterior foot pressure in the right and left feet and physique (height and body mass) were significant; however, the degree was very low (left foot vs. height: *r* = 0.20, *P* < 0.05; left foot vs. body mass: *r* = 0.18, *P* < 0.05; right foot vs. height: *r* = 0.22, *P* < 0.05; right foot vs. body mass: *r* = 0.18, *P* < 0.05). Even if a correlation coefficient is significant, a value under 0.2 is generally judged as having little relationship in terms of determination coefficient (*r*^2^). Hence, the effect of physique on the variables was not considered at the time of analysis.

### Statistical analysis

Intraclass correlation coefficients (ICC 1.1) of single measurement values in a one-way analysis of variance (ANOVA) model were calculated to examine inter-trial reliability for the ratio of anterior foot pressure in the right and left feet. Two-way ANOVA was used to test age differences and right/left differences for the variables. Because there were no sex differences for the variables [9], the combined data of boys and girls were used for the above analysis. If a significant difference was found, Tukey’s honestly significant difference test (HSD) was used for multiple comparisons. To examine interindividual differences, coefficients of variance, maxima, minima, and ranges were calculated. Pearson’s correlation coefficients were calculated to examine relationships between the right and left feet for the variables. An unpaired Student’s *t* test was used to analyze differences in variables between young children and adults, and a paired Student’s *t* test was used to analyze differences in variables between the right and left adult feet. The level of statistical significance was set at *P* < 0.05.

## Results

Intraclass correlation coefficients for the measured variables were 0.88 and 0.86 for the right and left feet, respectively. Table 2 shows basic statistics and test results according to age and right/left differences in each variable. Coefficients of variance, maxima, minima, and ranges for each age were 34.2 to 44.6, 57.0 to 83.0, 4.0 to 16.0, and 48.5 to 72.5, respectively. Coefficients of variance were highest in children aged 3.5 years and lowest in children 6.5 years. The ratio of anterior foot pressure was significantly larger in the right foot than in the left foot. With regard to age, anterior foot pressure was significantly larger in children aged over 4.5 years than in children aged 3.5 years. It was also significantly larger in children aged 6 and 6.5 years than in children aged 4 years. The effect size between the means of the right and left feet was 0.18. The larger the age difference was, the larger the effect size was (effect size = 0.44 to 0.78). Significant correlations were found between the right and left feet for all age groups (3.5 years, *r* = 0.65; 4 years, *r* = 0.58; 4.5 years, *r* = 0.78; 5 years, *r* = 0.69; 5.5 years, *r* = 0.66; 6 years, *r* = 0.65; 6.5 years, *r* = 0.82; entire study group, *r* = 0.70).

**Table 2:** Basic statistics and test results of age and right/left differences for the variables

		**3.5 years old**	**4 years old**	**4.5 years old**	**5 years old**	**5.5 years old**	**6 years old**	**6.5 years old**	**Total**	**Two-way ANOVA**				**Tukey’s HSD**	**Effect size**
											**Right-left**	**Age**	**Interaction**		
The ratio of anterior foot pressure in the left foot (%)	Mean	24.9	27.5	30.4	29.9	30.8	32.2	34.1	30.0	*F*-value	37.96*	7.23*	0.84	3.5<4.5,5.5,6,6.5	3.5 to 4.5: 0.44
	SD	11.1	10.4	11.2	11.6	11.2	11.5	11.7	11.4	*P*-value	0.00	0.00	0.54	6.5 4<6, 6.5	3.5 to 5: 0.47
	CV	44.6	37.9	36.9	38.8	36.3	35.8	34.2	38.0						3.5 to 5.5: 0.49
	Maximum	60.0	58.0	67.5	83.0	70.0	79.5	68.5	83.0						3.5 to 6: 0.62
	Minimum	6.0	8.5	9.5	11.0	5.5	11.0	16.0	5.5						3.5 to 6.5: 0.78
	Range	54.0	49.5	58.0	72.0	64.5	68.5	52.5	77.5						4 to 6: 0.49
The ratio of anterior foot pressure in the right foot (%)	Mean	27.3	28.5	32.0	33.2	32.6	34.6	36.5	32.1						4 to 6.5: 0.66
	SD	11.2	10.6	12.0	12.1	12.0	12.6	13.0	12.1						Left to right: 0.18
	CV	40.9	37.0	37.5	36.4	36.9	36.2	35.7	37.9						
	Maximum	60.0	57.0	73.5	70.5	77.5	81.5	67.0	81.5						
	Minimum	4.0	8.5	9.5	8.0	10.0	9.0	14.0	4.0						
	Range	56.0	48.5	64.0	62.5	67.5	72.5	53.0	77.5						

*P < 0.05. Because there were no sex differences for the variables, the analysis was conducted by considering boys and girls together. *CV*, coefficient of variance; *SD*, standard deviation.

The variables for the left and right feet of the adult controls were 45.6 ± 9.6 and 43.2 ± 10.1, respectively; the difference in variables between children and adults was statistically significant (left foot: *t* = 8.19, *P* < 0.05; right foot: *t* = 5.47, *P* < 0.05). When an adult’s value was 100% in both feet, a child’s value (6.5 years) corresponded to 74.7% in the left foot and 84.5% in the right foot. There were no significant differences between the right and left feet of adults (*t* = 1.03, *P* = 0.31). Coefficients of variance were 21.0 for the left foot and 23.4 for the right foot.

## Discussion

Matsuda *et al*. [9] reported that ICCs for anterior foot pressure in the right and left feet were 0.85 and higher in the second and third trials. This was consistent with the finding of our study, where ICCs for the variables were over 0.86 in the second and third trials. Therefore, the reliability of data used in this study was good.

This study examined age differences in anterior-posterior foot pressure ratio using the ratio of anterior foot pressure in each foot as a variable. The sample size was large enough to divide subjects into six-month categories. When children aged over 4.5 years and children aged 6 and 6.5 years were compared with children aged 3.5 years and children aged 4 years, respectively, the values for anterior foot pressure in each foot were significantly higher in the older age groups. The larger the age difference was, the larger the effect size was (effect size = 0.44 to 0.78). From these results, it can be concluded that the ratio of anterior foot pressure increases with age in young children. Kojima and Takemori [6] examined the effects of age on this variable in subjects aged 1.5 to 20 years and reported that the position of the center of foot pressure moved forward with age. In our study, the ratio of anterior foot pressure was smaller in young children than in adults. Ratios for young children aged 6.5 years corresponded to ratios of approximately 75% to 85% for adults. It can be concluded that the ratio of anterior foot pressure begins to increase from childhood and gradually approaches the adult ratio.

The anterior-posterior position of the center of foot pressure is located at approximately 40% of the foot length from the heel in adults; this position corresponds to the center of the longitudinal arch [8], and it is said that the postural stance is mechanically stable at this position [8]. Because instability while standing decreases and postural stability increases from childhood to adulthood [7,13,14], it is believed that an increase in the ratio of anterior foot pressure with age relates to an improvement in postural stability.

Matsuda *et al*. [2] examined the phenomenon of untouched toes during a two-year longitudinal study and reported that the number of untouched toes decreased every year in early childhood. The more forward the center of gravity is while standing, the greater is the toe pressure [8]. Therefore, an increase in the toe surface area touching the floor while standing directly affects the ratio of anterior foot pressure. In addition, the foot shape of young children, including the formation of the median longitudinal arch, changes significantly [15,16]. There may be relationships between changes in the shape of the foot, such as the formation of the median longitudinal arch and age change in the anterior-posterior foot pressure ratio. In future studies, it will be necessary to examine the relationships of both.

Usui *et al*. [7] examined changes in the anteriorposterior position of the center of foot pressure in children aged 3 to 12 years and reported that the position rapidly moved forward from 6 to 10 years. We also found that differences in anterior foot pressure ratio were larger between children aged 3.5 years and those aged 6 to 6.5 years and between children aged 4 years and those aged 6 to 6.5 years. These results suggest the possibility that the ratio of anterior foot pressure increases rapidly after 6 years of age. Investigation of changes in the anterior-posterior foot pressure after 6 years of age is a topic for future study.

Fujiwara [8] reported that the anterior-posterior position of the center of foot pressure was located at 30% to 60% of the foot length from the heel in adults aged 20 to 79 years, with large interindividual differences. Because the coefficients of variance, maxima, minima, and ranges were 34.2 to 44.6, 57.0 to 83.0, 4.0 to 16.0, and 48.5 to 72.5, respectively, for the ratio of anterior foot pressure in this study, it can be concluded that interindividual differences in young children are also large. The coefficients of variance were highest in children aged 3.5 years and lowest in children aged 6.5 years, and tended to decrease with age. In addition, they were higher in young children than in adults. It is to be inferred that individual differences in the ratio of anterior foot pressure decreases from early childhood to adulthood. Because young children have much less experience in standing than adults, the standing postures of young children may differ significantly between individuals. Age and interindividual differences in anterior-posterior foot pressure ratios were examined on the basis of cross-sectional data in this study; therefore, a longitudinal study is required to substantiate the findings of this study.

Correlation coefficients between the ratio of anterior foot pressure in the right foot and that in the left foot were not very strong. Furthermore, determination coefficients (*r*^2^) were less than 67%. Therefore, it can be inferred that the ratio differ slightly between the left and right foot.

The ratio of anterior foot pressure was slightly larger in the right foot than in the left foot (effect size = 0.18). It was reported that there were no right/left differences in the variables of foot pressure during movement [17,18]. Because the right/left difference in this study was small, it might be specific to the participants of this study. In addition, unlike the previous studies [17,18], in this study, foot pressure during static standing posture was measured. Hence, it is also considered that right/left differences are found in the variables of foot pressure during static standing posture. Heredity, environment, or incidental phenomena have previously been suggested as potential causes of right/left differences in human beings [19-21]. Because various factors relate to right/left differences, it is difficult to identify the cause of differences in this study. It was reported that children displaying right hand/foot dominance were more numerous than those with left hand/foot dominance or mixed hand/foot dominance from childhood [22]. Because the dominant hand/foot (right side) is used frequently in daily life, the right side of the body may be more developed than the left side. Right/left differences in organ position (for example, the heart) or organ weight are also considered as possible causes. In addition, because a right/left difference was not found in the adults evaluated in this study, the difference may be specific to young children. To clarify the cause of these differences and the time when these differences disappear, it would be necessary to examine the anterior-posterior foot pressure ratio in older adults.

In this study, a detailed analysis of the anterior-posterior foot pressure ratio was conducted. However, there is no present understanding of the clinical meaning of the difference of the value in the anterior-posterior foot pressure ratio. In future, this meaning will become clear through examination of the relationships between the anteriorposterior foot pressure ratio and factors such as standing posture, active level, body composition, and physical fitness (muscle strength, balance ability, and so on).

## Conclusions

This study examined age-related, interindividual, and right/left differences in anterior-posterior foot pressure ratio in 764 preschool children (364 boys and 400 girls) aged 3.5 to 6.5 years. The ratio of anterior foot pressure increased with age in young children. Interindividual differences in this ratio were large but tended to decrease with age. In addition, the ratio of anterior foot pressure was slightly larger in the right foot than in the left foot. The detailed actual condition regarding the anterior-posterior foot pressure ratio in young children can be obtained from the current results. These results will be useful for various studies, such as examining relationships between the anterior-posterior foot pressure ratio and factors, such as untouched toes, physical fitness, and exercise quantity.

## Abbreviations

ANOVA: Analysis of variance; ES: Effect size; HSD: Honestly significant difference test; ICC: Intraclass correlation coefficients.

## Competing interests

The authors declare that they have no competing interests.

## Authors’ contributions

SM performed the experiment, analysed the data, and wrote the manuscript. SD have been involved in drafting the manuscript and revising it. Both authors have read and approved the final manuscript.
